# Building a responsive teacher: how temporal contingency of gaze interaction influences word learning with virtual tutors

**DOI:** 10.1098/rsos.140361

**Published:** 2015-01-14

**Authors:** Hanju Lee, Yasuhiro Kanakogi, Kazuo Hiraki

**Affiliations:** 1 Department of General Systems Studies, University of Tokyo Komaba, Meguro, Tokyo 153-8902, Japan; 2 Core Research for Evolutional Science and Technology, Japan Science and Technology Agency, Chiyoda-ku, Tokyo 102-0075, Japan; 3 Graduate School of Education, Kyoto University, Kyoto, Japan

**Keywords:** pedagogical agent, temporal contingency, gaze interaction, educational technology, embodied artificial agent

## Abstract

Animated pedagogical agents are lifelike virtual characters designed to augment learning. A review of developmental psychology literature led to the hypothesis that the temporal contingency of such agents would promote human learning. We developed a Pedagogical Agent with Gaze Interaction (PAGI), an experimental animated pedagogical agent that engages in gaze interaction with students. In this study, university students learned words of a foreign language, with temporally contingent PAGI (live group) or recorded version of PAGI (recorded group), which played pre-recorded sequences from live sessions. The result revealed that students in the live group scored considerably better than those in the recorded group. The finding indicates that incorporating temporal contingency of gaze interaction from a pedagogical agent has positive effect on learning.

## 2. Introduction

Animated pedagogical agents are life-like virtual characters designed to enhance learning. These agents are receiving increasing attention as a new tool for computer-based education, supported by promising results from initial studies [1]. A wide variety of animated pedagogical agents have appeared; these agents range from realistic human forms in three-dimensional [2], to talking heads [3], animated parrots [4] and cartoon human characters [5]. Previous studies have examined a variety of features of such agents, including voice [4], appearance [6] and gender [7], to gauge their impact on learning. However, the knowledge of animated pedagogical agents is yet limited, and more research is needed to further identify the aspects of these agents that have potential to improve learning.

Social interaction of pedagogical agents is an area that requires more research. Developmental psychology literature suggests that human learning is influenced by social interaction. The ability to socially interact with others begins very early [8] and is thought to be crucial for learning general knowledge [9,10] and language [11,12]. Not surprisingly, children under 3 years of age learn less from screen media than from engaging in live social interaction with adults [13,14]; a phenomenon called the video-deficit effect [15].

While the cause of video deficit is not yet fully understood, several studies have found that providing live social interaction through screen media mitigates the effect [16,17]. For example, Nielsen *et al.* [17] demonstrated that when 2 year olds communicated with an experimenter through a closed circuit TV system, the children were as likely to succeed in imitating the experimenter's actions as the children who interacted directly with the experimenter.

Facilitating social interaction has been one of the central topics in pedagogical agents literature [1]. However, the approach has been largely concentrated on the animation quality of agents, such as liveliness of agent's gesture [18,19] and voice [4]. Recent developments in social neuroscience suggest the need for a more reciprocal approach, suggesting that social cognition may be fundamentally different when individuals are interacting with others rather than merely observing [20,21]. For example, Redcay *et al.* [22] showed that live interaction with a human experimenter, compared with viewing video recordings of the interaction, displayed greater activation in brain regions involved in social cognition and reward. In addition, Schilbach *et al.* [23] demonstrated that forming joint attention with a virtual character stimulates areas of the brain associated with social cognition, while avoiding joint attention recruited areas related to control of attention and eye movements.

Social interaction holds a distinct feature: temporal contingency. Indeed, human social cues involve a high level of temporal regulation. When humans communicate, gestures [24] and eye movements [25] become temporally coupled. Also, recent evidence suggests that such temporal regulation, or temporal contingency, also influences language learning. For example, Goldstein *et al.* [26] demonstrated that the immediate reactions of mothers to infant vocalizations or gestures facilitate speech production from infants. Therefore, this study focused on temporal contingency between the student and pedagogical agent.

Social interaction involves many domains, such as gesture, facial expression and voice tone. Gaze is one of the most well-described social cues [27], can be easily measured and be added to virtual characters with relative ease. Also, previous research from the field of developmental psychology indicates that gaze interaction is critical for the early stage of language learning [11,12,28]. For experimental purposes, we simplified gaze interaction into three key elements, mutual gaze, joint attention and gaze following, which are thought to play crucial roles in learning [11,12,28–30].

For learning material, we used foreign language vocabulary. While it is not a suitable teaching material for pedagogical agents to establish superiority over traditional teaching formats (e.g. books), we wanted to start with a material that adds less complication to experiment design. Adopting features for more advanced materials would propose another research topic, as we have to consider how to adopt each feature to fit the materials. In summary, our aim is to test the feature—temporal contingency—of the pedagogical agent and examine its effect *per se* as much as possible. To do so we started with a simple learning material, foreign language words.

This study is theoretically based on previous findings related to infants or children, but targets adults. Although it is necessary to note the differences between adult learning and child learning, learning unfamiliar words poses relatively similar demands to both children and to adults. Moreover, why the video deficit effect diminishes with age is under debate, and it would be interesting to see whether factors facilitating screen-media-learning in children could potentially impact adult learners.

In this study, we tested the hypothesis that the temporally contingent gaze interaction of animated pedagogical agents would enhance word learning. We developed an animated pedagogical agent capable of temporal contingent gaze interaction, called a Pedagogical Agent with Gaze Interaction (PAGI). PAGI simulates mutual gaze, gaze following, and joint attention with students while teaching foreign language words.

## 3. Material and methods

### 3.1 Participants

Thirty participants (seven women) were recruited from a subject pool at the University of Tokyo. Their mean age was 20.19 (s.d.=1.47) years. Participants were all native Japanese without Korean language experience. An additional five participants were not included in the sample owing to the failure of the eye-tracking system during the experiment, which caused the agent to malfunction. Participants were randomly assigned to either live (*n*=15; women=4; mean age=20.0) or recorded (*n*=15; women=3; mean age=20.4) group.

### 3.2 Design

A between-subjects yoked-condition design was used, in which participants learned Korean words with the temporally contingent agent (live group) or the recorded agent (recorded group). The live group was paired with the live-interacting pedagogical agent, and the recorded group with the agent replaying behaviour sequence recorded during live group sessions. Thus, the recorded group was provided with the same agent exhibiting the same behaviours in the exact sequence as the live group, except without temporal contingency.

### 3.3 The pedagogical agent with gaze interaction

The PAGI is an experimental animated pedagogical agent designed to teach Korean words to Japanese students. It is a three-dimensional male cartoon character voiced by a male Korean–Japanese bilingual speaker, lip-synced to the voice using predefined visemes.

**Figure 1.**
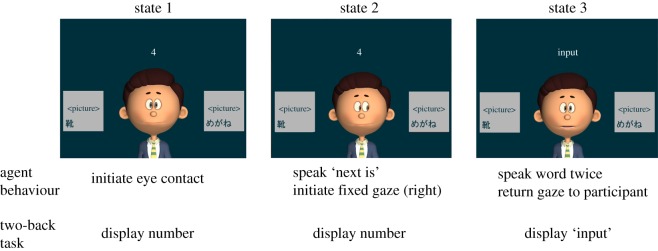
Gaze interaction scheme of PAGI.

PAGI started with an opening narration, explaining that he will be teaching Korean words, while gazing at participant's eyes, initiating eye contact (figure 1). After the narration, PAGI initiated the word learning phase. First, two pictures were presented (stage 1). PAGI waited for an eye contact and then shifted his gaze to the target picture. He then waited for the participant to follow his gaze and fixate on the target picture, and form joint attention. After joint attention was formed, PAGI returned his gaze to the participant and spoke a frame sentence (the first portion of the sentence leading to a target word, e.g. ‘this is’ or ‘next is’ in Japanese (stage 2)). Finally, PAGI spoke the target Korean word twice (stage 3). PAGI repeated stages 1–3 for each word.

In Stage 2, if the participant did not form the joint attention within 3 s time limit, PAGI looked at the participant and delivered attention-redirecting dialogue (‘Please follow my lead’) in Japanese. This was to mimic the behaviour of human tutor delivering attention-redirecting dialogue and to prevent the live group participants from taking advantage of the system and taking too much time to memorize each word. However, a pilot test revealed that for the replay group, the dialogue impaired the perceived reliability of the system, which is a confounding factor that could critically damage the experiment. Thus, recordings containing attention-redirection dialogues were not used for the recorded group. As a result, seven recordings were distributed to 15 recorded group participants.

### 3.4 Materials

All dialogues except target words were presented in Japanese. Korean nouns with less than four syllables were used for the lesson. Each word was presented with a corresponding picture and written Japanese word (figure 1). The word list was identical for all participants and was presented in the same sequence. The words were selected by a Korean–Japanese bilingual based on two criteria, low resemblance between the pair and familiarity to the general population (see the electronic supplementary material, Appendix A).

**Figure 2.**
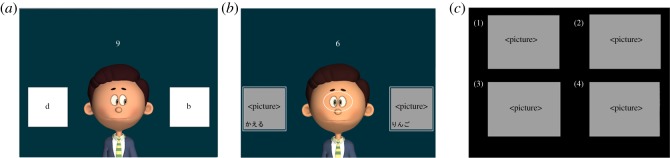
Selected frames from the stimuli: (*a*) the practice session, (*b*) the learning phase, PAGI state 1 (the white lines represent areas of interest and were not visible during the experiment), and (*c*) test phase. (The pictures are greyed-out due to copyright restrictions.)

A distracter picture was presented with each target picture to force gaze following. If only the target picture was presented, gaze following would be unnecessary. To avoid this, a random picture from the word list was simultaneously presented as a non-target word. As a result, to obtain the correct meaning of the word, participants needed to watch and follow PAGI's gaze. The target and distracter picture pairs were presented randomly to the left or right of PAGI (figure 2*b*). Participants learned 60 words, which were divided into two blocks of 30 words, with a 1 min rest between the blocks.

As the task was simple and repetitive, a pre-test revealed a ceiling effect, and participants reported that the difference between two conditions was too minor to be noticed. To solve these two problems, we employed a dual-task design using a digit span two-back task. A single-digit number appeared above PAGI and then was replaced by ‘input’, thus forcing participants to take their eye away from PAGI from time to time. This enabled participants to notice whether PAGI was responding to them or not. Participants were instructed to input the digit using a keyboard numpad while the two-back sign was showing ‘input’. When the answer was inputted during the input time window, the sign changed to blank to inform participants that their input was handled, regardless of its correctness. Key inputs made when the sign showed otherwise (a number or blank) were ignored. The two-back task was temporally synced to the word learning task; started and ended at the same time as each word (figure 1).

### 3.5 Apparatus

The eye-tracker (Tobii, Sweden) was integrated with a 17 inch LCD monitor, on which stimuli were displayed. A nine-point calibration was administered at the start of every block. A webcam placed under the eye-tracker focused on the participant's eyes to monitor the gaze interaction. For the test phase, a 17 inch CRT monitor was used. Sound stimuli were presented through two speakers (BOSE Media Mate II).

### 3.6 Procedure

When the participants arrived at the laboratory, each was led separately to a room and seated in front of a monitor. The experimenter told each participant that the purpose of the experiment was to assess how humans learn a foreign language and instructed them to engage in gaze interaction with the agent, by forming mutual gaze and following the agent's gaze. The experiment was divided into two phases: learning and test. In the beginning of the learning phase, each participant was given a practice session to get accustomed to PAGI and rehearse the digit span two-back task. The practice session was identical to the learning phase, except that instead of the Korean words, 10 English alphabet letters—from ‘a’ to ‘j’—were presented (figure 2*a*). Thus, each participant was given 10 trials (each alphabet counted as one trial) in the practice session. For the two-back task, participants were instructed to use any fingers of their preference, and to return all fingers to the starting position after each input; all fingers placed in line below numpad.

The test phase was carried out immediately after the learning phase. Four pictures were presented on the screen and a Korean word was verbally given (figure 2*c*). Participants were instructed to pick the picture that corresponded to the word. Participants used number keys 1–4 on a keyboard during the test to choose the picture. Participants were informed that there was no time limit during the test phase. The entire experiment lasted 25–30 min.

## 4. Results

**Figure 3.**
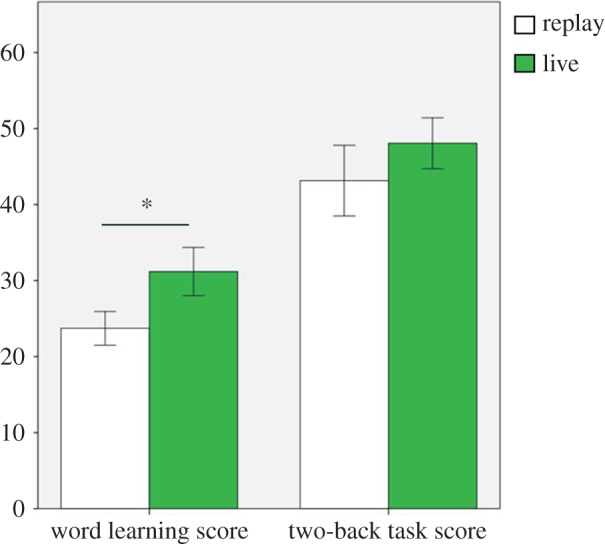
Mean of test results. Left: word learning. Right: two-back task. The asterisk indicates statistical significance. Error bars represent standard errors.

The test score was composed of the number of correct answers. Participants from both groups scored higher than the chance level; 15 words as the test phase consisted of 64 choice questions (live group: *t*_14_=10.193, *p*<0.001; recorded group: *t*_14_=7.572, *p*<0.001, one-sample *t*-test). The mean test scores differed significantly between the two groups (*t*_28_=3.372, *p*=0.002, *d*=1.24; figure 3).

The live group also performed better on the two-back task, with marginal significance (*t*_28_=3.046, *p*=0.095). This was expected as the two-back task was temporally synced to the word learning task, thus was also under temporal contingency effect, albeit less explicit than the main task.

As the experiment used dual-task design, the difference of attention allocation on two tasks may have affected the result (i.e. the live group assigned a larger proportion of cognitive resource on the word learning task). The difference could not be compared between the two groups, however, as both tasks were affected by temporal contingency. Nonetheless, analysis on each group showed no significant correlations between the test scores and two-back scores. Also, group difference of test scores cannot be explained by attention allocation as the live group scored better on both tasks.

Generally, memory task performance increases when participants are given more time, and the time spent on the learning phase was not controlled in our experiment. To assess the influence of the time spent on learning, we examined the relationship between participants' learning time and test scores. The duration of the learning phase did not differ significantly between the two groups (live group: *M*=368.7 s, recorded group: *M*=352.8 sec; *t*_28_=1.391, *p*>0.1). There was no significant correlation between learning phase duration and test scores. These analyses revealed that time spent on learning did not significantly affect the test scores.

Because the attention-redirecting dialogue was provided only for the live group, it may have influenced the result. To assess this, we conducted an analysis using the live group data. The overall number of received attention-redirecting dialogues was small (*M*=1.27); seven participants did not receive any, four received one, two received two and two received four. As the distribution of attention-redirecting dialogue counts were outside of the limits of normality (standardized skewness coefficient greater than 2), a nonparametric procedure, Spearman's rho was used for the analysis. The correlation between the number of received dialogues and test scores was not significant (*r*=0.439, *p*>0.1, Spearman's rho). In addition, we observed no significant difference in test scores between participants who received the attention-redirecting dialogue and those who did not (*t*_13_=1.298, *p*>0.2). This provides some evidence that the attention-redirecting dialogue did not significantly affect the result.

The eye-tracking data were gathered using commercial software (Tobii Studio, Sweden). As eye-tracking data is inherently noisy, we conducted strict pre-selection of data. The samples containing less than 50 valid fixations (data points classified as fixation inside the area of interest that does not contain missing gaze points and was longer than 100 ms—which is argued to be the minimum fixation duration; Tobii Fixation Filter [31] was used for fixation classification) were excluded from the analysis for statistical validity. As a result, three participants from the live group and five from the replay group were excluded for eye-tracking data analysis.

The eye-tracking data analysis revealed no immediate difference between the two groups. Reaction time for mutual gaze and joint attention was calculated by measuring the time elapsed from PAGI's gaze shift and subsequent fixation on the target object (mutual gaze: PAGI, joint attention: target picture). There was no significant difference in the reaction time between groups either in mutual gaze (live group: *M*=852.33 ms, recorded group: *M*=828.46 ms, *p*>0.8) or joint attention (live group: *M*=896.99 ms, recorded group: *M*=780.43 ms, *p*>0.1). To analyse participants' commitment to each gaze interaction, the proportion of each gaze behaviour was assessed. The proportion of direct gaze was calculated as the total duration that the participant fixated on PAGI while PAGI was looking at the participant/total duration of PAGI looking at participant; joint attention as total duration that the participant fixated on the target picture while PAGI was looking at the picture/total duration of PAGI looking at the picture. There was no significant difference between the two groups, for mutual gaze (live group: *M*=0.291, recorded group: *M*=0.180, *p*>0.1) or joint attention (live group: *M*=0.437, recorded group: *M*=0.290, *p*>0.1). None of these factors were correlated with the test scores.

**Figure 4.**
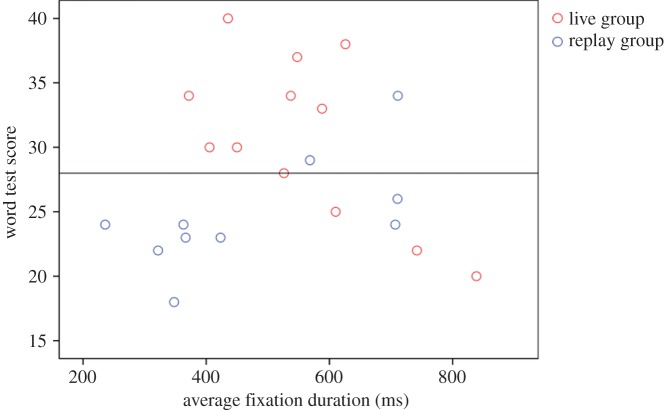
Scatterplot illustrating the relationship between word test scores and average fixation duration. The horizontal line represents the median test score. Eight participants were not included in the eye-tracker data analysis (but included in test scores) due to lack of valid data, as explained in ‘Results’.

To further evaluate the differences in visual search patterns, the average fixation duration was measured. The fixations on PAGI and pictures were subjected to the analysis. There was no significant difference between the groups (live group: *M*=556.54 ms, recorded group: *M*=475.52 ms, *p*>0.2), and there was no significant correlation with the test scores. However, additional analysis revealed that a certain time window of fixation duration was related to higher test scores (figure 4). The average fixation duration of the higher scoring group (split by the test score median) was inside 350∼750 ms, with only two samples outside the 400∼700 ms window. Indeed, participants with average fixation duration within 400∼700 ms scored significantly better (*n*=11, *M*=31.55) than those with shorter or longer gaze duration (*n*=11, *M*=24.64, *t*_20_=3.086, *p*=0.006). The distribution of samples regarding the duration window differed significantly between the two groups; 9 out of 12 members of the live group were inside the time window compared with 2 out of 10 from the replay group (Fisher's exact test, two-tailed, *p*=0.03).

Interestingly, the correlation between the test scores and the fixation duration was in the opposite direction (live group: *r*=−0.626, *p*=0.03, recorded group: *r*=0.644, *p*=0.045). The replay group was more likely to have a fixation average shorter than 350 ms (Fisher's exact test, two-tailed, *p*=0.029), which appears to reflect the need for more frequent visual search. As for the live group, excessive fixation duration was related to lower test scores, which we initially believed to be the consequence of attention gaps. As attention gaps should have increased the likelihood of receiving attention-redirecting dialogues, we assessed if there was a correlation between the fixation duration average and the attention-redirecting dialogue count, but it did not reach significance (*r*=−0.041, *p*>0.1, Spearman's rho).

## 5. Discussion

The present study demonstrated that temporally contingent gaze interaction improves the word learning with animated pedagogical agents. Our study supports the importance of live social interaction on human learning and extends this to computer-based education.

Because the attention-redirecting dialogue was presented only for the live group, we initially suspected that the dialogue might have influenced the result. However, our auxiliary analysis suggests that the influence was negligible. This seems at a glance to contradict with previous finding of D'Mello *et al.* [32], who reported that the use of attention-redirecting dialogue improved learning of high school biology. However, they also reported that the effect was confined to deep reasoning, and the effect was not found in overall learning, especially in directly transferred knowledge. As attention-redirecting dialogue is strongly related to boredom or drift of attention, its effect is understandably influenced by complexity of learning material and the length of lesson. The facts that the learning material of our experiment was relatively simple and short should have minimized the effect of the attention-redirecting dialogues, as shown in our result.

If the attention-redirecting dialogue did not improve learning, it remains unclear why the temporal contingency of gaze interaction had such a significant effect. One possible explanation is that lack of temporal contingency induced larger cognitive load by inflicting the need for more frequent visual search; the replay group participants had to constantly check for the agent's cues whereas the live group participants could progress on their own terms. The analysis of the fixation duration average showed that the live group was more likely than the replay group to have fixation average inside the time window of 400∼700 ms which was linked to higher test scores, and the replay group was more likely to have shorter fixation average. While fixation duration cannot always be directly linked to cognitive processes, in regard to our experimental task, shorter fixation durations can be linked to greater devotion towards visual search. Therefore, it can be assumed that temporal contingency reduced extraneous cognitive load, contributing to less burden for visual search.

The analysis also showed that for the live group, excessive fixation duration was related to lower test scores. This is probably attributable to a lack of motivation. The participants may have thoughtlessly followed the instruction by exhibiting gaze behaviours, but did not invest full attention to word learning. If this was the case, fixation duration may be used to counter low attention in real world settings, especially for students with low motivation. To this end, how fixation duration is affected by motivation is an interesting research topic and requires future research.

Another major factor influencing the result may be social presence. In social agency theory, social cues of pedagogical agents prime a feeling of social partnership in the learner, which leads to deeper cognitive processing during learning, and results in a more meaningful learning outcome [1,19]. Previous studies suggest that the social agency of pedagogical agents have an impact on learner motivation [6,33], and the design of these agents could alter the motivational outcomes [6,18]. In addition, recent evidence from social neuroscience implies that social cues from a virtual agent—direct gaze and socially relevant facial expression—recruit brain regions related to emotional processing [34]. The temporal contingency of gaze interaction may have influenced perceived social agency, affecting the motivation of participants. While D'Mello *et al.* [32] reported gaze reactivity of a pedagogical agent did not produce motivational outcomes, the gaze interaction in the study was limited to attention-redirecting dialogue which the agent vocalized when student's gaze was away for a specific time. PAGI's interacting behaviours were more reciprocal and real time, thus may have yielded greater social presence, resulting in more motivational outcomes. More research is needed, however, to confirm that the temporal contingency of pedagogical agents facilitate learning motivation.

The most important topic deferred for future work is ‘follow- and lead-in’ of joint attention and its effect on learning. In this study, joint attention was initiated by the pedagogical agent, which put participants in a follow-in position. While the interaction still proved beneficial, previous studies indicate that lead-in joint attention may be favourable to follow-in joint attention. Schilbach *et al.* [23] found that lead-in gaze interaction activates the brain area related to the reward system, which implies having learners lead gaze interaction could enhance motivational outcomes. In addition, evidence from developmental psychology suggests that lead-in joint attention has more benefits than follow-in joint attention in early language development [35]. Therefore, adding lead-in joint attention to the agent may lead to enhanced learning outcomes.

This study also proposes the merit of using artificial agents in the field of developmental psychology as a tool for assessing human-to-human interactions. The merits of using virtual environments in psychological experiments has been discussed [36] and has been successfully implemented in social neuropsychology [23,34]. In this study, by using an artificial agent, we had strict control over the temporal contingency, which would have been difficult for a human experimenter. Therefore, we believe artificial agents have potential as experimental tools. For example, one construct that can benefit forthwith from using artificial agents is the video deficit effect. Currently, the most common practice when examining the video deficit effect is to use human experimenters as a counterpart to video stimuli [14,37,38]. There are two limitations to this experimental design. First, strict control over variables cannot be ensured. Second, the granularity of the experiment is limited by the precision of human perception. For example, the latency of mutual gaze cannot be controlled in milliseconds. We believe artificial agents open new possibilities for assessing subtle human behaviours, precisely and cost-efficiently.

In conclusion, our experiment has led us to conclude that temporal contingency of gaze interaction is a key feature in the improvement of the effectiveness of animated pedagogical agents. Our data suggest that temporal contingency should be considered when designing animated pedagogical agents. Furthermore, our methodology proposes the merit of using artificial agents in the field of developmental psychology to overcome the limits of previous experimental methods involving human experimenters.

## Supplementary Material

Appendix A: The list of learning materials used in the study

## Data accessibility

The learning materials are uploaded as electronic supplementary material, Appendix A.

## References

[1] Moreno R, Mayer RE, Spires HA, Lester JC 2001 The case for social agency in computer-based teaching: do students learn more deeply when they interact with animated pedagogical agents? Cogn. Instr. 19, 177–213. (doi:10.1207/s1532690xci1902_02)

[2] Graesser AC, Lu SL, Jackson GT, Mitchell HH, Ventura M, Olney A, Louwerse MM 2004 AutoTutor: a tutor with dialogue in natural language. Behav. Res. Methods Instr. Comput. 36, 180–192. (doi:10.3758/bf03195563) 10.3758/bf03195563 15354683

[3] Moundridou M, Virvou M 2002 Evaluating the persona effect of an interface agent in a tutoring system. J. Comput. Assisted Learn. 18, 253–261. (doi:10.1046/j.0266-4909.2001.00237.x)

[4] Atkinson RK, Mayer RE, Merrill MM 2005 Fostering social agency in multimedia learning: examining the impact of an animated agent's voice. Contemp. Educ. Psychol. 30, 117–139. (doi:10.1016/j.cedpsych.2004.07.001)

[5] Craig SD, Gholson B, Driscoll DM 2002 Animated pedagogical agents in multimedia educational environments: effects of agent properties, picture features, and redundancy. J. Educ. Psychol. 94, 428–434. (doi:10.1037//0022-0663.94.2.428)

[6] Baylor AL 2011 The design of motivational agents and avatars. Educ. Technol. Res. Dev. 59, 291–300. (doi:10.1007/11423-011-9196-3)

[7] Kim Y, Baylor AL, Shen E 2007 Pedagogical agents as learning companions: the impact of agent emotion and gender. J. Comput. Assist. Learn. 23, 220–234. (doi:10.1111/j.1365-2729.2006.00210.x)

[8] Grossmann T, Johnson MH 2007 The development of the social brain in human infancy. Eur. J. Neurosci. 25, 909–919. (doi:10.1111/j.1460-9568.2007.05379.x) 17331189 10.1111/j.1460-9568.2007.05379.x

[9] Csibra G, Gergely G 2009 Natural pedagogy. Trends Cogn. Sci. 13, 148–153. (doi:10.1016/j.tics.2009.01.005) 19285912 10.1016/j.tics.2009.01.005

[10] Tomasello M, Carpenter M, Call J, Behne T, Moll H 2005 Understanding and sharing intentions: the origins of cultural cognition. Behav. Brain Sci. 28, 675–735. (doi:10.1017/s0140525x05000129) 16262930 10.1017/S0140525X05000129

[11] Morales M, Mundy P, Delgado CEF, Yale M, Messinger D, Neal R, Schwartz HK 2000 Responding to joint attention across the 6-through 24-month age period and early language acquisition. J. Appl. Dev. Psychol. 21, 283–298. (doi:10.1016/s0193-3973(99)00040-4)

[12] Brooks R, Meltzoff AN 2005 The development of gaze following and its relation to language. Dev. Sci. 8, 535–543. (doi:10.1111/j.1467-7687.2005.00445.x) 16246245 10.1111/j.1467-7687.2005.00445.x PMC3640988

[13] Kuhl PK, Tsao FM, Liu HM 2003 Foreign-language experience in infancy: effects of short-term exposure and social interaction on phonetic learning. Proc. Natl Acad. Sci. USA 100, 9096–9101. (doi:10.1073/pnas.1532872100) 12861072 10.1073/pnas.1532872100 PMC166444

[14] Krcmar M, Grela B, Lin K 2007 Can toddlers learn vocabulary from television?: An experimental approach. Media Psychol. 10, 41–63. (doi:10.1080/15213260701375652)

[15] Anderson DR, Pempek TA 2005 Television and very young children. Am. Behav. Sci. 48, 505–522. (doi:10.1177/0002764204271506)

[16] Troseth GL, Saylor MM, Archer AH 2006 Young children's use of video as a source of socially relevant information. Child Dev. 77, 786–799. (doi:10.1111/j.1467-8624.2006.00903.x) 16686801 10.1111/j.1467-8624.2006.00903.x

[17] Nielsen M, Simcock G, Jenkins L 2008 The effect of social engagement on 24-month-olds' imitation from live and televised models. Dev. Sci. 11, 722–731. (doi:10.1111/j.1467-7687.2008.00722.x) 18801128 10.1111/j.1467-7687.2008.00722.x

[18] Baylor AL, Kim S 2009 Designing nonverbal communication for pedagogical agents: when less is more. Comput. Hum. Behav. 25, 450–457. (doi:10.1016/j.chb.2008.10.008)

[19] Mayer RE, DaPra CS 2012 An embodiment effect in computer-based learning with animated pedagogical agents. J. Exp. Psychol. Appl. 18, 239–252. (doi:10.1037/a0028616) 22642688 10.1037/a0028616

[20] Schilbach L, Timmermans B, Reddy V, Costall A, Bente G, Schlicht T, Vogeley K 2013 Toward a second-person neuroscience. Behav. Brain Sci. 36, 393–414. (doi:10.1017/s0140525x12000660) 23883742 10.1017/S0140525X12000660

[21] Anders S, Heinzle J, Weiskopf N, Ethofer T, Haynes JD 2011 Flow of affective information between communicating brains. Neuroimage 54, 439–446. (doi:10.1016/j.neuroimage.2010.07.004) 20624471 10.1016/j.neuroimage.2010.07.004 PMC3081064

[22] Redcay E, Dodell-Feder D, Pearrow MJ, Mavros PL, Kleiner M, Gabrieli JDE, Saxe R 2010 Live face-to-face interaction during fMRI: a new tool for social cognitive neuroscience. Neuroimage 50, 1639–1647. (doi:10.1016/j.neuroimage.2010.01.052) 20096792 10.1016/j.neuroimage.2010.01.052 PMC2849986

[23] Schilbach L, Wilms M, Eickhoff SB, Romanzetti S, Tepest R, Bente G, Shah NJ, Fink GR, Vogeley K 2010 Minds made for sharing: initiating joint attention recruits reward-related neurocircuitry. J. Cogn. Neurosci. 22, 2702–2715. (doi:10.1162/jocn.2009.21401) 19929761 10.1162/jocn.2009.21401

[24] Shockley K, Santana MV, Fowler CA 2003 Mutual interpersonal postural constraints are involved in cooperative conversation. J. Exp. Psychol. 29, 326–332. (doi:10.1037/0096-1523.29.2.326) 10.1037/0096-1523.29.2.326 12760618

[25] Richardson DC, Dale R, Kirkham NZ 2007 The art of conversation is coordination—common ground and the coupling of eye movements during dialogue. Psychol. Sci. 18, 407–413. (doi:10.1111/j.1467-9280.2007.01914.x) 17576280 10.1111/j.1467-9280.2007.01914.x

[26] Goldstein MH, King AP, West MJ 2003 Social interaction shapes babbling: testing parallels between birdsong and speech. Proc. Natl Acad. Sci. USA 100, 8030–8035. (doi:10.1073/pnas.1332441100) 12808137 10.1073/pnas.1332441100 PMC164707

[27] Emery NJ 2000 The eyes have it: the neuroethology, function and evolution of social gaze. Neurosci. Biobehav. Rev. 24, 581–604. (doi:10.1016/s0149-7634(00)00025-7) 10940436 10.1016/s0149-7634(00)00025-7

[28] Striano T, Chen X, Cleveland A, Bradshaw S 2006 Joint attention social cues influence infant learning. Eur. J. Dev. Psychol. 3, 289–299. (doi:10.1080/17405620600879779)

[29] Senju A, Csibra G 2008 Gaze following in human infants depends on communicative signals. Curr. Biol. 18, 239–252. (doi:10.1016/j.cub.2008.03.059) 10.1016/j.cub.2008.03.059 18439827

[30] Okumura Y, Kanakogi Y, Kanda T, Ishiguro H, Itakura S 2013 The power of human gaze on infant learning. Cognition 128, 127–133. (doi:10.1016/j.cognition.2013.03.011) 23672983 10.1016/j.cognition.2013.03.011

[31] Olsson P 2007 Real-time and offline filters for eye tracking. Masters thesis, KTH Royal Institute of Technology, Stockholm, Sweden Retrieved from DiVA (diva2:573446).

[32] D'Mello S, Olney A, Williams C, Hays P 2012 Gaze tutor: a gaze-reactive intelligent tutoring system. Int. J. Hum. Comput. Stud. 70, 377–398. (doi:10.1016/j.ijhcs.2012.01.004)

[33] Heidig S, Clarebout G 2011 Do pedagogical agents make a difference to student motivation and learning? Educ. Res. Rev. 6, 27–54. (doi:10.1016/j.edurev.2010.07.004)

[34] Schilbach L, Wohlschlaeger AM, Kraemer NC, Newen A, Shah NJ, Fink GR, Vogeley K 2006 Being with virtual others: neural correlates of social interaction. Neuropsychologia 44, 718–730. (doi:10.1016/j.neuropsychologia.2005.07.017) 16171833 10.1016/j.neuropsychologia.2005.07.017

[35] Tomasello M, Farrar MJ 1986 Joint attention and early language. Child Dev. 57, 1454–1463. (doi:10.1111/j.1467-8624.1986.tb00470.x) 3802971

[36] Blascovich J, Loomis J, Beall AC, Swinth KR, Hoyt CL, Bailenson JN 2002 Immersive virtual environment technology as a methodological tool for social psychology. Psychol. Inquiry 13, 103–124. (doi:10.1207/s15327965pli1302_01)

[37] Roseberry S, Hirsh-Pasek K, Parish-Morris J, Golinkoff RM 2009 Live action: can young children learn verbs from video? Child Dev. 80, 1360–1375. (doi:10.1111/j.1467-8624.2009.01338.x) 19765005 10.1111/j.1467-8624.2009.01338.x PMC2759180

[38] Krcmar M 2010 Can social meaningfulness and repeat exposure help infants and toddlers overcome the video deficit? Media Psychol. 13, 31–53. (doi:10.1080/15213260903562917)

