# Supplementary material for: Building a responsive teacher: how temporal contingency of gaze interaction influences word learning with virtual tutors
Source: R Soc Open Sci. 2015 Jan 14;2(1):140361. doi: 10.1098/rsos.140361 (PMC4448798; doi:10.1098/rsos.140361)
Supplement: Appendix A: The list of learning materials used in the study [file rsos140361supp1.docx]

Appendix A

Stimuli in the presented order

Korean/Japanese/English

ha-neul-saek/ aiiro/ azure

ppal-gang-saek/ aka/ red

ga-eul/ aki/ autumn

so-geum-jaeng-i/ amenbou/ water strider

pa-rang/ ao/ blue

me-ttu-gi/ batta/ grasshopper

ju-hwang/ daidai/ orange color

mulbanggae/ gengoro/ diving beetle

pa-ri/ ha-e/ fly(insect)

hoe-saek/ haiiro/ grey

bom/ haru/ spring

jeot-ga-rak/ hasi/ chopsticks

baem/ hebi/ snake

bi-haeng-gi/ hikouki/ airplane

chaek/ hon/ book

gyeo-ul/ huyu/ winter

gae/ inu/ dog

ui-ja/ isu/ chair

mo-gi/ ka/ mosquito

gae-gu-ri/ kaeru/ frog

geo-ul/ kagami/ mirror

jong-i/ kami/ paper

ji-u-gae/ keshigomu/ eraser

na-mu/ ki/ tree

ssal/ kome/ rice

ja-dong-cha/ kuruma/ car

sin-bal/ kutsu/ shoes

sa-seum-beol-le/ kuwagata/ stag beetle

ttal-gi/ ichigo/ strawberry

dol-go-rae/ iruka/ dolphin

kong/ mame/ bean

an-gyeong/ megane/ glasses

cho-rok/ midori/ green

bok-sung-a/ momo/ peach

bo-ri/ mugi/ barley

ji-ne/ mukade/ centipede

bo-ra/ murasaki/ purple

bae/ nasi/ asian pear

yeo-reum/ natsu/ summer

go-yang-i/ neko/ cat

sa-gwa/ ringo/ apple

saeng-seon/ sakana/ fish

jeop-si/ sara/ plate

won-sung-i/ saru/ monkey

seol-tang/ satou/ sugar

so-geum/ sio/ salt

sa-ja/ sisi/ lion

sut-ga-rak/ supoon/ spoon

ja-du/ sumomo/ plum

cham-sae/ suzume/ sparrow

gan-jang/ syoyu/ soy sauce

gye-ran/ tamago/ egg

yang-pa/ tamanegi/ onion

mu-dang-beol-le/ tentoumushi/ ladybug

hak/ toki/ crane

ho-rang-i/ tora/ tiger

chaek-sang/ tsukue/ table

bu-chae/ uchiwa/ folding fan

ja-jeon-geo/ zitensya/ bicycle

ko-kki-ri/ zyou/ elephant
